# In vivo comparison of enteric capsule shells composed of different HPMCAS grades after simultaneous intake by dual MRI labeling and pharmacokinetics

**DOI:** 10.1016/j.ijpx.2026.100666

**Published:** 2026-09-15

**Authors:** Michael Grimm, Ruben Lau, Fiona Mankertz, Robin Bülow, Felix Morof, Delphine Nombret, Camille Dumont, Vincent Jannin, Mladen Vassilev Tzvetkov, Werner Weitschies

**Affiliations:** a Department of Biopharmaceutics and Pharmaceutical Technology, University of Greifswald, 17489 Greifswald, Germany; b Department of Diagnostic Radiology and Neuroradiology, University Hospital Greifswald, 17475 Greifswald, Germany; c Department of Radiology, University Hospital Tuebingen, 72076 Tuebingen, Germany; d Department of General Pharmacology, University Hospital Greifswald, 17487 Greifswald, Germany; e Capsugel France SAS, 68000 Colmar, France

**Keywords:** Enteric hard capsule, Hydroxypropyl methyl cellulose acetate succinate (HPMCAS) grades, Magnetic resonance imaging (MRI), Dual labeling, Manganese gluconate, Saliva, Stable isotope, Caffeine, Intraindividual variability

## Abstract

Different grades of hypromellose acetate succinate (HPMCAS) differ in their pH dependent dissolution behavior, which is of key relevance when used for enteric coating or gastroresistant capsule shells. By use of specific grades it could be possible to tailor intestinal release in terms of timing and localization. A crossover design might be the gold standard for comparison of two dosage forms but intraindividual variability in gastrointestinal transit conditions still limit robust comparability. In the present study, two prototype capsule shells made of HPMCAS grade L and M, were compared with reference Capsugel® Enprotect® capsules made of grade H. For avoidance of intraindividual variability affecting the comparison of two dosage forms with a limited group of subjects, an approach with an internal standard should be used. Thus, reference capsules and the two types of test capsules were administered by healthy young volunteers in fasted state in a 2-arm crossover study. Test capsule and reference capsule were always administered together allowing for an internal reference. To enable differentiation in magnetic resonance imaging (MRI), the reference and test capsules contained different amounts of black iron oxide. The test capsules additionally contained manganese gluconate. The capsules also contained different types of ^13^C-labeled caffeine to act as distinguishable pharmacokinetic markers. Different amounts of iron oxide led to distinguishable artifacts in TRUFI sequences. Manganese gluconate led to a bright cloud in VIBE sequences when test capsule disintegrated, allowing for very sensitive detection of opening and interesting visualization of small intestinal spreading after release. Reference HPMCAS grade H capsules mainly disintegrated in ileum, while test capsules disintegrated in more proximal regions of small intestine or in terms of HPMCAS grade L even in stomach already. Utilization of an internal standard with dual labeling by dosing test and reference simultaneously was a relevant improvement in study design. HPMCAS grade M is a robust alternative to HPMCAS grade H if faster and more proximal disintegration is desirable.

## Graphical abstract

**Figure:**
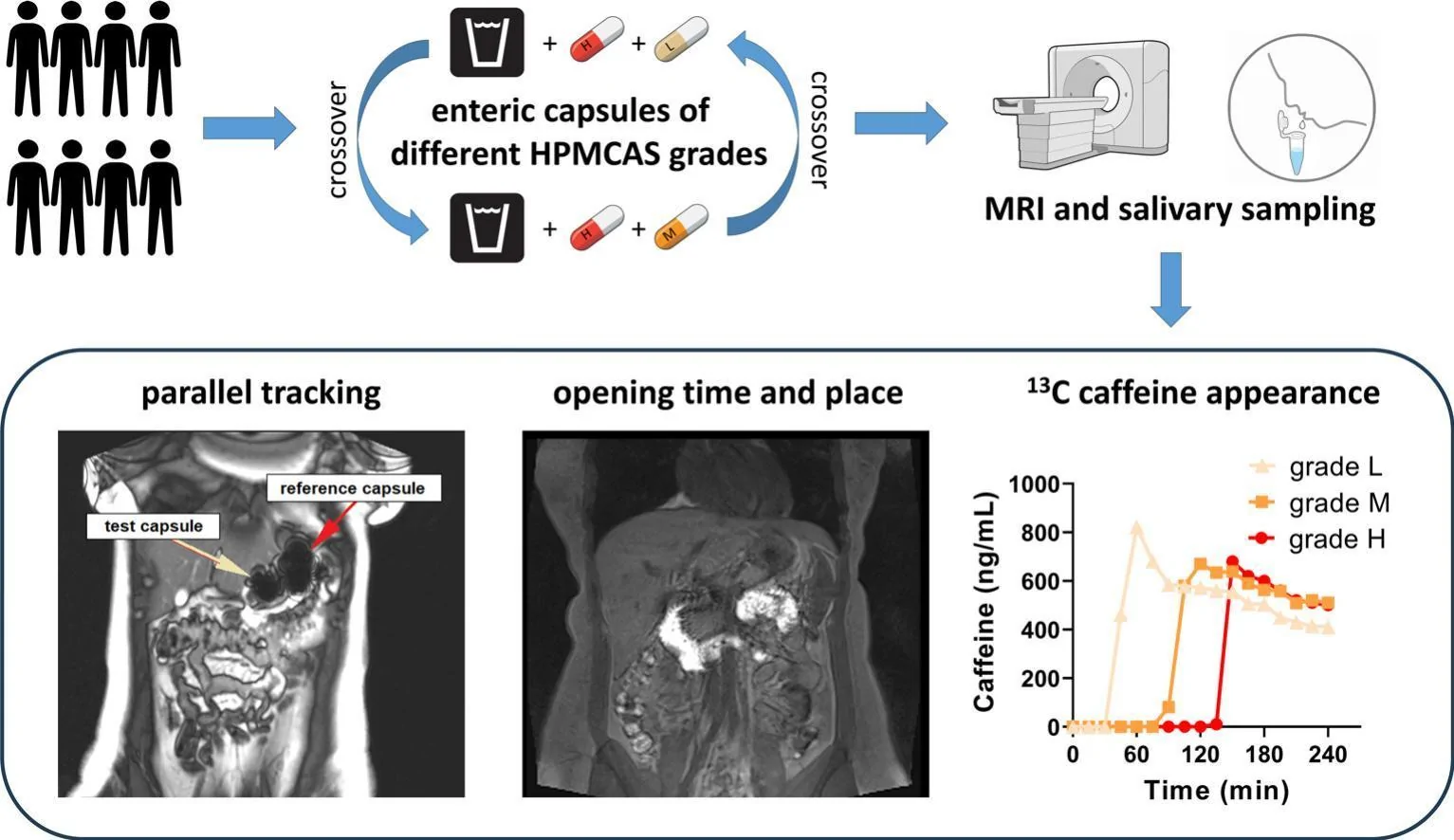

## 1 Introduction

Ready-to-fill enteric hard capsules gained more and more attention during the last years. Several gastro-resistant hard capsule products are available utilizing different enteric polymers such as hypromellose (HPMC) in combination with methacrylic acid (Eudracap® enteric), HPMC in combination with hypromellose phthalate (AR-CAPS® Acid Resistant capsules) and HPMC combined with hypromellose acetate succinate (Capsugel® Enprotect® capsules) (Rump et al., 2022; Grimm et al., 2023). Hypromellose acetate succinate (HPMCAS) is available in specific grades referring to high (H), medium (M) and low (L) substitution grade of succinate groups on the HPMC chain. These different substitution grades of acidic groups lead to differences in pH dependent solubility. According to literature, HPMCAS grade H dissolves at pH ≥ 6.8, grade M at pH ≥ 6.0 and grade L at pH ≥ 5.5 (Sarabu et al., 2020). Keeping in mind the physiological pH profiles in small intestines, it should be possible to tailor the release of an enteric dosage form containing HPMCAS at different regions of the small intestine. This way, release site in the intestines could be adjusted to the needs of the respective API using the same enteric capsule platform, by variegating the grade of HPMCAS.

Considering the intraindividual variability in gastrointestinal transit of such monolithic dosage forms, it could be beneficial to compare such tailored vehicles after simultaneous administration, using an imaging technique to assure site-specific release and discrimination of co-administered dosage forms. Although, data on intraindividual variability of dosage form transit are scarce and effects remain speculative, it is very likely that intraindividual variability could interfere the comparability in a typical crossover design. Therefore, it would be statistically more robust, if more than one system could be investigated in parallel. Thus, when comparing the in vivo performance of two dosage forms with each other, an internal standard is desirable. Since in vivo evaluations of dosage forms often require costly techniques, the simultaneous administration can further increase efficiency of studies.

The in vivo behavior of dosage forms can be evaluated by several methods including imaging and other diagnostic tools like pharmacokinetic marker substances. The investigation of disintegration time points or release from dosage forms using pharmacokinetic labels can be performed by blood sampling, but salivary sampling might be beneficial by being noninvasive, mostly inexpensive and being able to safely allow for higher temporal resolution in repetitive measurements. For this salivary tracer technique mainly paracetamol (acetaminophen) or caffeine are reported as labels (Senekowitsch et al., 2022).

Caffeine can be used in various isotope labeled versions and is already known to allow for simultaneous and distinguishable evaluation of beginning release from simultaneously taken oral dosage forms, since chemically they behave the same but remaining distinguishable in LC-MS/MS (Mark et al., 2024; Schiller et al., 2005). Although pharmacokinetic labels offer distinguishability of release and pharmacokinetics of simultaneously administered dosage forms, they do not allow for information about localization of disintegration and release, which is of main interest in terms of enteric or other site specific dosage forms. Information about localization in GI tract needs an imaging technique to evaluate transit and disintegration, opening or release. To increase robustness and sensitivity the imaging can also be combined with pharmacokinetic labels.

One of the methods that has been frequently used in the past to track dosage forms and their disintegration is gamma-scintigraphy (Großmann et al., 2025). A variety of tracers can be used for the scintigraphic labeling of pharmaceutical dosage forms. These include compounds of ^99^ᵐTc, ^153^Sm, ^171^Er, and ^111^In (Steingoetter et al., 2003; Akbar et al., 2024b). Due to the different radiation energies of these elements, they enable the simultaneous tracking of multiple dosage forms or the concurrent tracking of liquids and solid dosage forms. But availability and stability of radioactive labels, necessary imaging equipment, risks associated with ionizing radiation and regulatory hurdles limit the applicability of scintigraphy for dosage form tracking. Moreover, an anatomic reference image for orientation in GI tract is missing in scintigraphy. This is crucial for robust investigation of the disintegration site of an enteric dosage form due to high morphological variability which cannot be estimated from outside (Grimm et al., 2024).

Another option for dosage form evaluation in vivo is Magnetic Resonance Imaging (MRI) which offers good soft tissue contrast and anatomical orientation in high resolution, various contrast options, while avoiding ionizing radiation. Thus, MRI has been frequently utilized for dosage form evaluation in the past (Senekowitsch et al., 2022; Weitschies and Wilson, 2011; Akbar et al., 2024a). Nonetheless, studies and methodologies for the tracking of two or more simultaneously administered distinguishable pharmaceutical dosage forms in parallel are scarce. Chaddock et al. and Mark et al. reported tracking of several non-disintegrating capsules in parallel, but these have been no pharmaceutical dosage forms aimed to deliver their filling. These capsules were made out of an insoluble polymer and filled with gadolinium contrast agent to investigate object transit for evaluation of motility patterns (Chaddock et al., 2014; Mark et al., 2024). Moreover the method seemed not to allow for distinguishability of the individual capsules.

There are only a few other studies which investigated the transit of more than one distinguishable and simultaneously administered dosage form using MRI. In the study by Schiller et al. several capsules which were administered simultaneously in a specific sequence could be distinguished in T2 weighted MRI by a unique pattern and number of watery gel spheres inside a solid triglyceride matrix (Schiller et al., 2005). But intravenous application of butylscopolamine hydrobromide was necessary to sufficiently paralyze intestinal motility for avoidance of movement artifacts. Such small internal structures inside the capsules would not have been distinguishable during natural movement and another method would be needed if undisturbed transit and dosage form performance should be investigated.

A combination of common tracking labels e.g. black iron oxide labeled powder for T2* sensitive imaging and dried pineapple pieces for T1 weighted imaging (Grimm et al., 2019) in two dosage forms should allow for distinguishable evaluation after simultaneous intake. In a study by Großmann et al. an oil filled capsule and a tablet labeled with black iron oxide were successfully investigated in parallel (Großmann et al., 2025). Nonetheless the method required a 3 T MRI scanner with sufficient spatial resolution and capsules were filled with a liquid filling in a capsule-in-capsule design. This limits applicability for other more representative dosage forms and combination with other necessary excipients. Dried pineapple pieces could also be utilized as contrasting filling, but large pieces are necessary, leaving not much space for other fillings of interest and their residual moisture as well as their batch-dependent signal also limit their applicability (Grimm et al., 2019).

Gadolinium based contrast agents would theoretically work well and have been used in the past (Steingoetter et al., 2003), but for representative dosage forms which should release their payload, these substances seem inappropriate nowadays. Gadolinium should only be used after thorough risk-benefit assessment and as a drug substance it also provokes ethical and regulatory issues.

Recently the approved food additive manganese gluconate gained more attention as MRI contrast agent for evaluation of dosage form performance (Akbar et al., 2024b; Grimm et al., 2024). It is a safe excipient and can be included in powder formulations facilitating representative solid oral dosage forms and combination with other excipients. Manganese gluconate acts as a positive T1-weighted MRI contrast agent. Upon dissolution in aqueous media, Mn^2+^ ions shorten the longitudinal relaxation time (T1) of surrounding water protons. As these water protons and their preceding spins are mainly responsible for MRI signal in intestinal lumen, this results in increased signal intensity and a bright contrast enhancement on T1-weighted images in a concentration dependent manner. For this mechanism to be effective, manganese gluconate must dissolve and release Mn^2+^ into an aqueous environment, so that the unpaired electron spins of these ions can interact with the excited spins of the water protons in the hydration shell of the respective ions. The local water availability therefore strongly influences the detectability of the contrast effect. Thus, additional labels like black iron oxide are still needed for robust detectability of the intact dosage form in the intestines before opening due to insufficient amount of water protons inside the dry dosage form. Additionally, a salivary tracer like caffeine can be added to the mixture allowing for simultaneous pharmacokinetic evaluation of different dosage forms in parallel if isotope labeled versions are used (Akbar et al., 2024b; Grimm et al., 2024).

For the aim to compare two simultaneously administered dosage forms, the combination of black iron oxide, manganese gluconate and different caffeine variants would therefore allow for internal standard and reduce the necessary amount of study arms.

Thus, this study had two objectives. First, an in vivo comparison of enteric capsule shells made out of HPMCAS grade H as reference, and HPMCAS grade M and HPMCAS grade L test capsules should be made. Moreover, a novel dual labeling together with pharmacokinetic marker should be established, to allow for simultaneous and distinguishable evaluation of at least two dosage forms by imaging and pharmacokinetics in parallel.

## 2 Materials and methods

### 2.1 Study materials

The administered capsules were filled comparable to previous studies with caffeine, iron oxide, superdisintegrant and mannitol based filling powder. But in this study, the amounts of black iron oxide were varied and a novel marker was included to allow simultaneous administration and subsequent evaluation of transit and disintegration of test and reference capsules in parallel. Details on used materials and excipients can be found in Table 1. Powder mixtures were prepared by use of a Turbula shaker mixer T2F (Willy A. Bachhofen AG, Germany) and filled manually and individually on a precision balance Sartorius AX623 (Sartorius Lab Instruments GmbH & Co. KG, Germany).

**Table 1:** Materials and excipients used for study capsules.

Ingredient	Producer/Distributor
Capsugel® Enprotect® size 0 HPMCAS grade H	Capsugel France SAS, Colmar, France
Capsugel® prototype capsules size 0 HPMCAS grade L	Innovaform® Accelerator, Capsugel France SAS, Colmar, France
Capsugel® prototype capsules size 0 HPMCAS grade M	Innovaform® Accelerator, Capsugel France SAS, Colmar, France
Black iron oxide E172	Caesar & Loretz GmbH, Hilden, Germany
Manganese gluconate	Dr. Paul Lohmann GmbH & Co. KGaA, Germany
^13^C_3_- labeled caffeine	Eurisotop - A Cambridge Isotope Laboratory Company, Cambridge, USA
^13^C_1_- labeled caffeine	Eurisotop - A Cambridge Isotope Laboratory Company, Cambridge, USA
Croscarmellose, Ph.Eur.	JRS Pharma GmbH & Co. KG, Rosenberg, Germany
Mannitol, Ph.Eur.	Caesar & Loretz GmbH, Hilden, Germany
Silicon dioxide, Ph.Eur.	Fagron GmbH & Co. KG, Barsbüttel, Germany

The resulting reference Capsugel® Enprotect® capsules (HPMCAS grade H) of size 0 were filled with a mixture of 17.9 mg black iron oxide, 25 mg ^13^C_3_ labeled caffeine, croscarmellose and standard filling powder of mannitol and colloidal silicon dioxide. Test capsules (HPMCAS grades L or M) of size 0 were filled with 2.2 mg black iron oxide, 25 mg ^13^C_1_ labeled caffeine, 125 mg manganese gluconate, croscarmellose and standard filling powder of mannitol and colloidal silicon dioxide as well. Capsule weight amounted to 390 ± 2 mg for HPMCAS grade H reference capsules, 435 ± 1 mg for HPMCAS grade L test capsules and 436 ± 1 mg for HPMCAS grade M test capsules. Test capsules have been slightly heavier due to higher bulk density of included manganese gluconate.

Simultaneously administered test and reference capsule have been distinguishable due to different amounts of iron oxide, which led to differently sized artifacts in TRUFI sequences (True Fast Imaging with Steady-State Precession). Manganese gluconate led to a bright “cloud” in T1 weighted VIBE sequences (Volumetric Interpolated Breath-hold Examination) when test capsule disintegrated. This way, further differentiation of opening was possible, since only test capsules would produce this specific signal in T1 weighted sequences. Details on imaging aspects can be found in the subsequent chapters *Experimental design* and *Magnetic Resonance Imaging sequences*. Furthermore, the different isotope labeled caffeine species offered distinguishable and sensitive evaluation of release.

### 2.2 Study participants

Eight healthy young volunteers (4 males, 4 females) were included in the study. They had a mean (± SD) age of 24.8 ± 4.5 years and a mean (± SD) BMI of 23.3 ± 2.9 kg/m^2^. Written informed consent was obtained after interested subjects had been checked for inclusion and exclusion criteria such as metallic implants, non-removable piercings, gastrointestinal impairments (operations, diseases) eating disorders, claustrophobia or pharmacotherapy with any other drug than hormonal contraceptives. In case of female subjects, pregnancy was precluded by negative urine pregnancy test. Subjects were informed about their rights and duties regarding possible incidental findings and had the choice to refuse the disclosure. All risks resulting from study participation including commuting accidents have been covered by dedicated insurances. Furthermore, an appropriate expense allowance for study participation was provided.

### 2.3 Experimental design

To investigate and compare the different HPMCAS based capsule shells a study with 2 arms was performed. In each study arm, a Capsugel® Enprotect® size 0 reference capsule made of HPMCAS grade H was applied together with a test capsule made of HPMCAS grade L (study arm A) or HPMCAS grade M (study arm B). Thus, subjects always took two capsules (reference + test) together. Subjects were blinded for test capsule type. Administration was performed with 240 mL of water at room temperature after at least 10 h overnight fasting. Moreover, subjects had to abstain from caffeine for 24 h and non-caloric fluids 1 h before each capsule administration.

Capsule transit and opening were evaluated by MRI and caffeine based salivary tracer technique, which are described in the following paragraphs. Before intake of the capsules, a reference MRI and blank saliva sample were taken. The intake of the capsules was defined as *t* = 0 min. MRI was performed in an interval of 15 min for up to 240 min. Subjects were allowed to leave the MRI scanner in between the measurements, but movements were highly restricted to avoid influences on motility due to physical activity. Thus, subjects have been sitting on a chair and were allowed to read or hear music.

MRI evaluation could be stopped prematurely, if disintegration of both capsules could be assured in two consecutive measurements. Sampling of saliva was performed by self-depended drooling into 1.5 mL micro tubes (Sarstedt AG & Co. KG, Nürnbrecht, Germany). Salivary sampling was also performed in an interval of 15 min directly after MRI measurement. Even if MRI evaluations were stopped, salivary sampling was always continued until 240 min. Intake of foods and beverages was prohibited until the end of the examination.

The study was conducted according to the guidelines of the Declaration of Helsinki, and approved by the Ethics Committee of the University Medicine of Greifswald (ethical protocol No. BB 115/24a). Moreover, the study was registered at the German Register for Clinical trials under DRKS00035518.

### 2.4 Salivary sample preparation and evaluation of caffeine pharmacokinetics

The analytical method, encompassing both salivary sample preparation and subsequent analysis, complied with the FDA Guidance for Industry on “Bioanalytical Method Validation”. A comprehensive description of the method is also available in previous publications (Rump et al., 2022; Grimm et al., 2023). At each time point, approximately 1 mL of saliva was collected and stored at −80 °C until the end of the sampling period (240 min). For analysis, samples were thawed, centrifuged (15 min at 18,000*g*), and precipitated by adding 200 μL of a solution containing 94% acetonitrile and 6% formic acid to 100 μL of saliva. The samples were then refrozen, thawed once more, and centrifuged again under the same conditions.

The LC-MS/MS system consisted of an Shimadzu Nexera LC-20 (Shimadzu, Duisburg, Germany) coupled to a API4000 QTRAP triple quadrupole mass spectrometer (AB Sciex, Darmstadt, Germany) equipped with a Turbo V™ electrospray ionization source. Data acquisition and processing were performed using validated Analyst 1.7 software (AB Sciex, Darmstadt, Germany). The lower limit of quantification (LLOQ) for ^13^C_1_ labeled and ^13^C₃ labeled caffeine was 5 ng/mL, referring to 15 ng/mL in undiluted saliva. The first measurement exceeding this concentration in undiluted saliva was defined as “caffeine appearance,” from which appearance times (AT) were calculated as described below.

### 2.5 Magnetic resonance imaging sequences

**Fig. 1:**
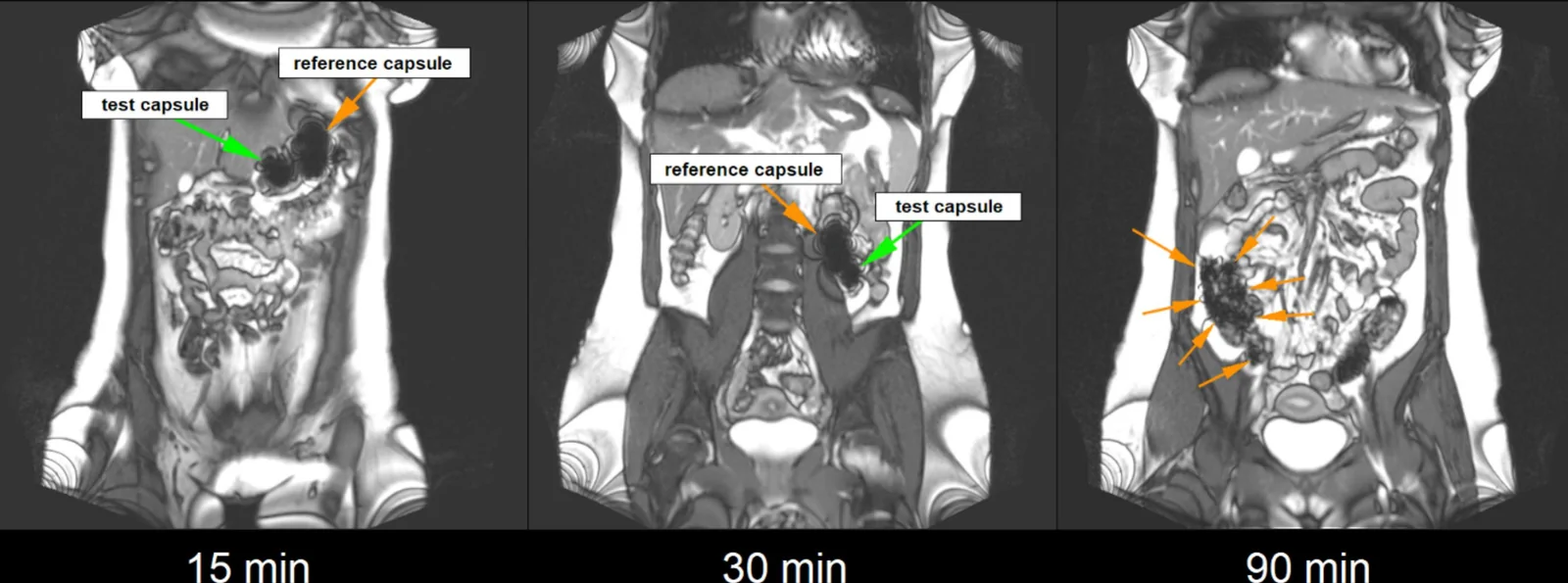
Exemplary coronal TRUFI images with differently sized and distinguishable susceptibility artifacts of reference capsule (orange) and test capsule (green) for simultaneous tracking and evaluation of disintegration. (For interpretation of the references to colour in this figure legend, the reader is referred to the web version of this article.)

**Fig. 2:**
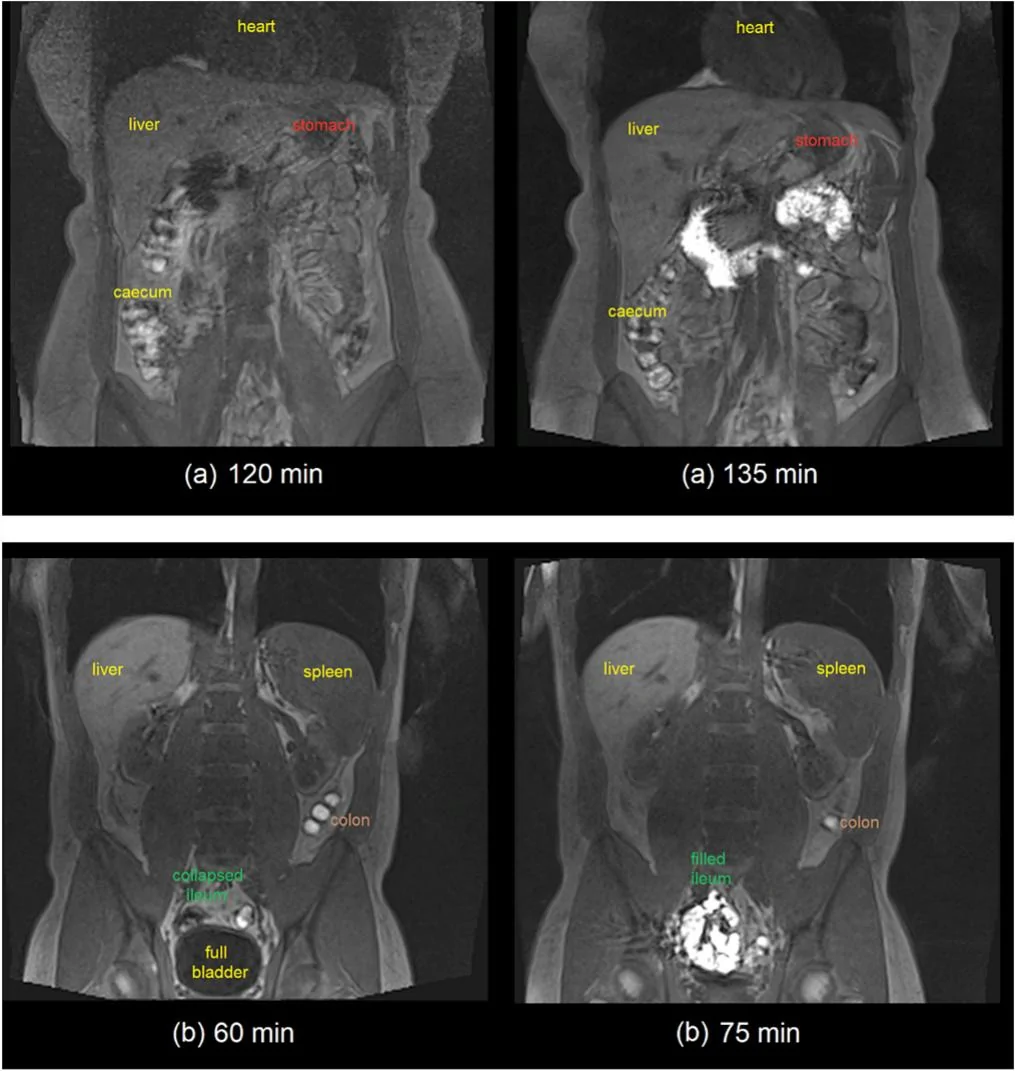
Bright clouds of released manganese from enteric capsules in coronal T1 weighted VIBE images before (left) and after opening (right) in two exemplary subjects with disintegration in duodenum (a) and ileum (b) within the same slice respectively.

Magnetic resonance imaging (MRI) investigations were conducted using a Siemens MAGNETOM Avanto 1.5 T MR scanner (Siemens Healthcare, Erlangen, Germany) at the Institute of Diagnostic Radiology and Neuroradiology, University Hospital Greifswald. All scans were performed with subjects in the supine position (lying on their back, head oriented forward). To monitor the two susceptibility artifacts, a T2 weighted TRUFI sequence was employed. Variations in the iron oxide content produced susceptibility artifacts of differing sizes, which could be distinctly visualized and differentiated using the TRUFI sequence, as illustrated in Fig. 1. Additionally, T1 weighted VIBE sequences with fat saturation were acquired to sensitively assess the opening of the test capsules. Upon rupture, the manganese gluconate was rapidly solubilized in the intestinal fluids, resulting in a hyperintense signal cloud in the VIBE sequences as illustrated in Fig. 2.

To minimize motion artifacts, participants were instructed to hold their breath for up to 25 s during each TRUFI acquisition and up to 18 s during each VIBE acquisition. Subjects only have to lie motionless during these breath holds. In between measurements, they were allowed to move slightly or leave the scanner.

Only coronal image slices were obtained throughout the imaging procedures. Details on sequence parameters can be found in Table 2.

**Table 2:** Sequence parameter for MR imaging.

	TRUFI	VIBE
**Slice thickness**	5 mm	3 mm
**Interslice gap**	0 mm	0 mm
**Repetition Time (TR)**	3.4 ms	3.72 ms
**Time to Echo (TE)**	1.43 ms	1.4 ms
**Flipangle**	53°	25°
**Matrix**	486 × 512	256 × 238
**Field of view (FOV)**	500 mm	400 mm
**Voxel size**	0.0048 mL	0.0073 mL

### 2.6 Image analysis

Horos Viewer Versions 3.3.1 and 3.3.6 (The Horos Project) were used for image evaluation. The position in gastrointestinal tract and integrity of the capsules was evaluated manually in the TRUFI image sets from a respective time point, while the respective VIBE sequence was evaluated with regards to appearance of hyperintense signal. Two independent and trained observers took part in the evaluation. In cases of discordant interpretations, ambiguous findings were resolved through discussion until consensus was achieved. The integrity of the capsule shell was assessed based on the size and morphology of the characteristic susceptibility artifacts. The presence of multiple small artifacts, alterations in artifact size, or changes in their geometry were interpreted as indicative of capsule filling dispersion. For dispersion to occur, substantial disintegration of the capsule is required. Consequently, the dispersion of the filling has previously been considered a reliable surrogate marker for capsule disintegration. However, it is recognized that visible dispersion typically lags behind the detection of the pharmacokinetic marker caffeine in saliva when disintegration takes place in the small intestine (Rump et al., 2022; Sager et al., 2019a). Exemplary images are given in Fig. 1.

Since the amount of iron oxide was reduced in test capsules to achieve a distinguishable much smaller but still trackable artifact, disintegration was most often harder to evaluate in TRUFI sequences since no visible dispersion could be obtained. The artifact of the test capsule most often just disappeared and did sometimes not allow for determination of gastrointestinal localization of disintegration in T2 weighted imaging. Thus, the appearance of hyperintense signal of manganese solution in specific regions was further used to sensitively evaluate disintegration of test capsule and its previous localization by the T1 weighted VIBE sequences. For visualization of manganese spreading in gastrointestinal tract as a model for water soluble drug substance, maximum intensity projections (MIP) were used e.g. in Fig. 7 and Fig. 8. The amount of stacked slices was chosen variably according to best visibility.

### 2.7 Capsule evaluation criteria

MRI was used to evaluate the dispersion time of capsule filling as well as gastric residence time and gastrointestinal localization of visible dispersion.

Salivary ^13^C_1_-caffeine appearance time of test capsules and ^13^C_3_-caffeine appearance time of reference capsules was assessed from salivary pharmacokinetics and exceedance of LLOQ. The gastric residence time and the dispersion time as observed via MRI, as well as the salivary caffeine appearance time were calculated as the mean of the first time point at which gastric emptying, dispersion or caffeine appearance was detected and the preceding time point. The region of the gastrointestinal tract in which the capsule was located at the time of the detected dispersion was assessed as the gastrointestinal localization of dispersion, sometimes referred as “site of dispersion”, “site of initial disintegration” or “disintegration compartment” in the past (Grimm et al., 2023; Grimm et al., 2019; Grimm et al., 2024). Other authors called it “location of disintegration” respectively (Varum et al., 2022).

The dispersion time after gastric emptying was calculated by subtracting the gastric residence time from dispersion time. In some publications, these parameters were referred as “Time to Disintegration after GE” (Sager et al., 2019a; Wilding et al., 1992) or “intestinal transit time of the capsules until disintegration” (ITT_D) (Rump et al., 2022).

The Appearance time was subtracted from Dispersion time to calculate the so-called Lag time, the time between observed dispersion of capsule filling to appearance of caffeine in saliva. This value can also be negative, if dispersion in MRI is observed after caffeine is already present in saliva. The smaller the absolute value of the lag time is, the better is the concordance between imaging and pharmacokinetics.

### 2.8 Statistical analysis

Dispersion times, caffeine appearance times and gastric residence times are given as mean ± standard deviation in continuous text and tables. Individual data for dispersion time and appearance time can further be found in Fig. 5. The statistical evaluations were performed using Excel 2019, GraphPad Prism 5 and OriginPro 8.5.1. Gaussian distribution was tested by Shapiro-Wilk and Kolmogorov-Smirnov normality tests. Both tests needed to allow acceptance of normal distribution, otherwise non-parametric testing was performed.

The parameters of reference HPMCAS grade H capsules were compared with HPMCAS grade L or HPMCAS grade M test capsules within each study arm. Moreover, reference capsules (both HPMCAS grade H) were compared with each other between the study arms to check for robustness of conventional crossover design. Also test capsules (HPMCAS grade M and L) where compared between study arms. Due to study design with test and reference in the same application as well as crossover design for repetitive measurement of a second reference/test-combination, all data are paired. For statistical comparison a Wilcoxon matched-pairs signed rank test with Bonferroni correction for 4 multiple tests (test A vs. reference A, test B vs. reference B, reference A vs. reference B, test A vs. test B) was used.

The dispersion time and appearance time of each capsule was compared individually to evaluate trends regarding method sensitivity and differences in capsule shell disintegration characteristics. Again, a Wilcoxon matched-pairs signed rank test with Bonferroni correction for 4 multiple tests was used.

To compare the sensitivity between iron oxide labeling and manganese gluconate labeling for evaluation of disintegration in MRI, the lag time between visual dispersion and salivary caffeine appearance was further statistically compared between all test capsules (iron oxide labeling) and all reference capsules (manganese labeling) by a paired non-parametric Wilcoxon signed rank test.

All differences were accepted significant if *p* < 0.05.

## 3 Results

All subjects completed the study successfully, and no adverse effects related to the study procedures were reported. All saliva samples and MRI images were suitable for evaluation. The labeling of the capsules with different amount of iron oxide allowed discrimination between test and reference capsule by size of susceptibility artifact in TRUFI in most cases. Only in a few images the small artifact was not detectable at one time point while re-appearing at the following image acquisition. This was observed when both capsules were lying close together in a relatively empty stomach and the large artifact of the reference capsule “overshadowed” the smaller one.

The evaluation of disintegration by iron oxide was also possible. The disintegration of the reference HPMCAS grade H capsules with higher iron oxide content was typically detectable by dispersion of artifacts, whereas the smaller artifacts of HPMCAS grade L and grade M test capsules filled with less iron oxide, tend to disappear completely after opening. Exemplary tracking and dispersion in T2 weighted TRUFI sequence based on iron oxide is depicted in Fig. 1.

In addition to the iron oxide labeling, the detection of opening of the test capsules was evaluated by appearance of hyperintense areas of dissolved manganese gluconate. This turned out to work even better than searching for the disappearance of the small iron oxide artifact. This way the dispersion time and localization of the test capsules could robustly be evaluated in T1 weighted sequences. Exemplary images are given in Fig. 2.

**Fig. 3:**
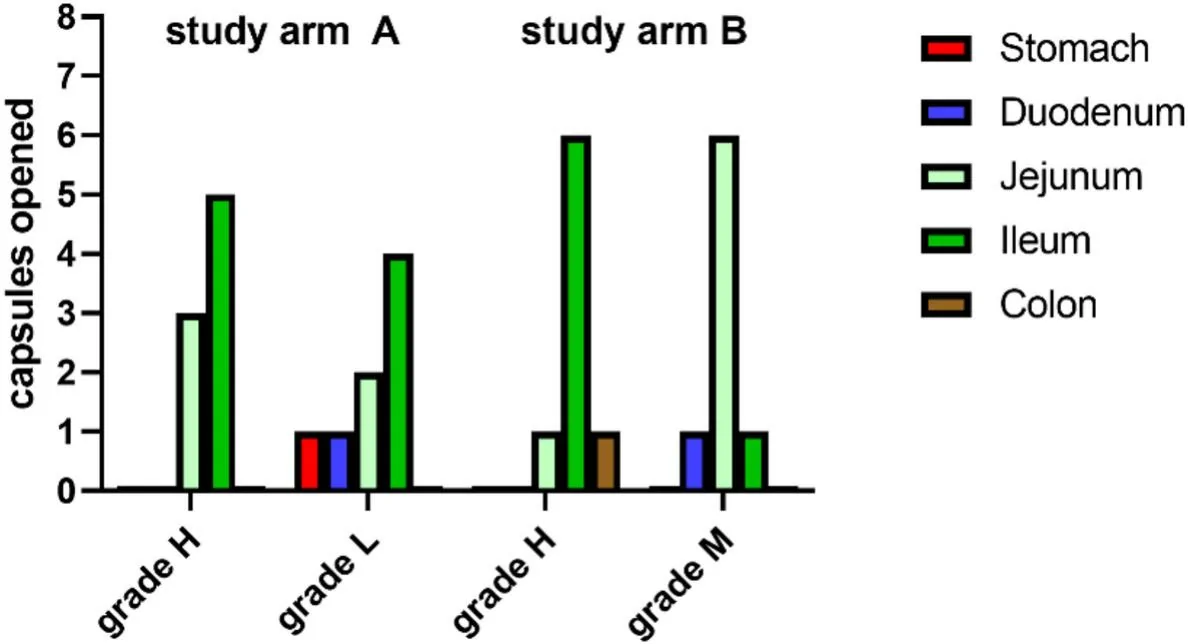
Gastrointestinal localization of visible filling dispersion from HPMCAS capsules of different grades administered simultaneously in arm A or B respectively (*n* = 8 each).

The combination of iron oxide tracking and manganese gluconate appearance allowed for evaluation of gastrointestinal localization of visible filling dispersion. Results are shown in Fig. 3.

5 of 8 and 6 of 8 reference Capsugel® Enprotect® HPMCAS grade H capsules opened in ileum in both study arms with rare opening events in jejunum respectively. In one subject even the colon was reached intactly, but this subject also showed a rapid intestinal transit from stomach to colon within 45 min, leaving no time for the capsule to open in small intestine.

The test capsules made of HPMCAS grade L and M generally showed opening in more proximal regions of the gastrointestinal tract. One HPMCAS grade L even disintegrated in the stomach already. This was well distinguishable as can be seen in Fig. 7. Detailed parameters on transit and opening from imaging can further be found in Table 3. Individual data can be found in Supplementary file in Tables S1, S2, S3 and S4.

**Table 3:** Transit and opening parameter of tested capsules (mean ± SD, Min-Max, [median], *n* = 8, or *n* = 7 marked with asterisk *) with significant difference according to *p* < 0.05 by Wilcoxon matched-pairs signed rank test with Bonferroni correction in study arm A (^A^) or B (^B^).

Parameter	Grade H	Grade L	Grade H	Grade M
Gastric Residence	43 ± 38 min 7.5–112.5 min [30 min]	35 ± 31 min* 7.5–97.5 min [23 min]	43 ± 48 min 7.5–142.5 min [23 min]	43 ± 45 min 7.5–127.5 min [23 min]
Dispersion Time	128 ± 39 min 67.5–187.5 min [135 min] ^A^	66 ± 36 min 22.5–112.5 min [53 min] ^A^	109 ± 42 min 52.5–157.5 min [105 min] ^B^	64 ± 40 min 37.5–127.5 min [45 min] ^B^
Dispersion Time after GE	84 ± 28 min 45–120 min [75 min] ^A^	34 ± 28 min* 0–90 min [30 min] ^A^	66 ± 28 min 15–105 min [68 min] ^B^	21 ± 14 min 0–30 min [30 min] ^B^
Caffeine Appearance Time	105 ± 39 min 52.5–157.5 min [113 min] ^A^	62 ± 36 min 22.5–112.5 min [53 min] ^A^	94 ± 41 min 37.5–142.5 min [90 min] ^B^	62 ± 37 min 37.5–127.5 min [45 min] ^B^

**Fig. 4:**
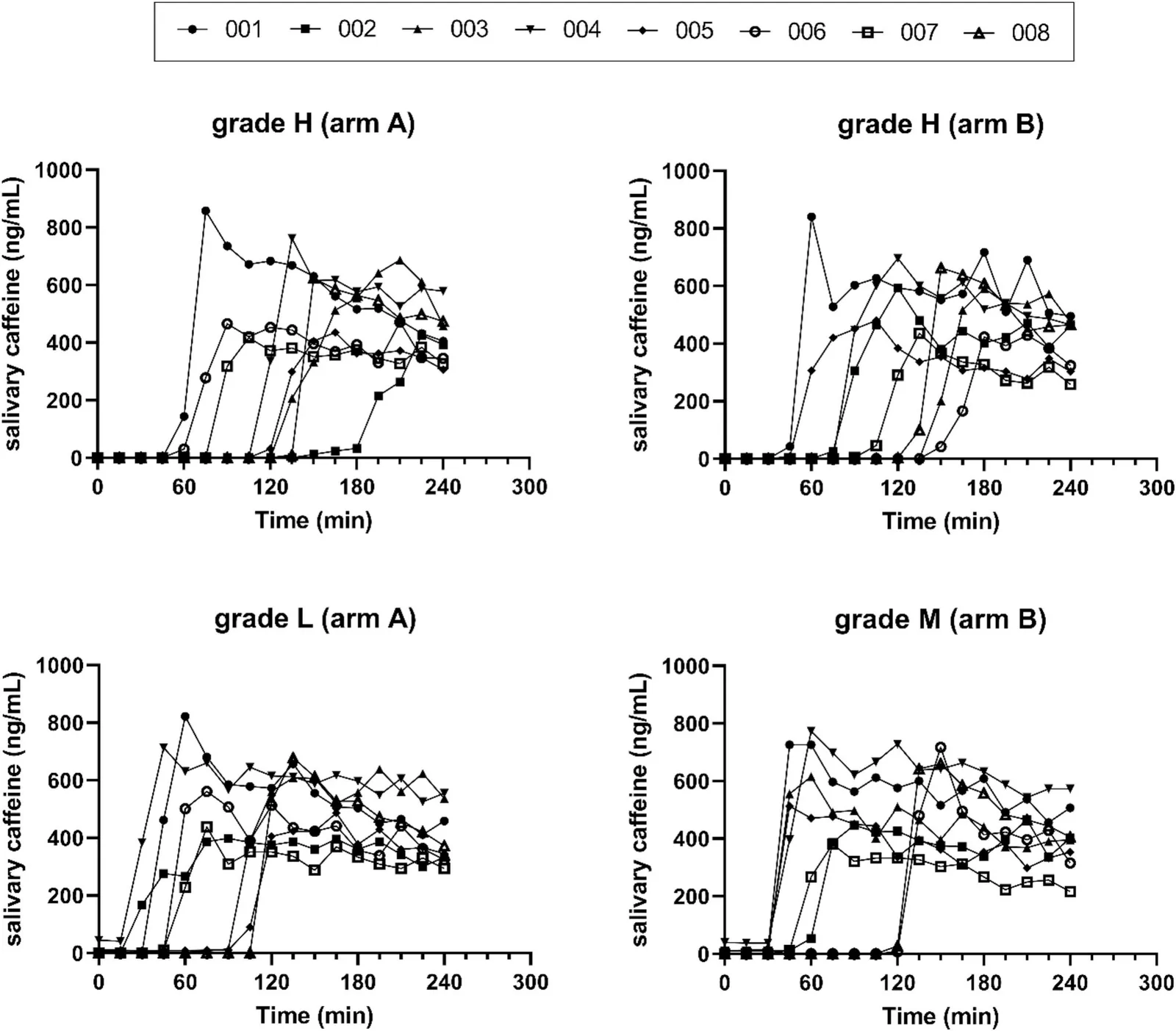
Salivary caffeine profiles of study arm A (left) and study arm B (right) for ^13^C_3_- labeled caffeine from reference Enprotect® capsules made of HPMCAS grade H and ^13^C_1_- labeled caffeine from test capsules made of HPMCAS grade L and M respectively (*n* = 8 each).

In addition to imaging, isotope labeled caffeine was used as an independent pharmacokinetic marker for capsule opening. Due to different amount of ^13^C in the caffeine of test and reference capsule filling, both profiles were distinguishable in parallel. Individual caffeine profiles can be seen in Fig. 4.

From the salivary caffeine pharmacokinetics, the appearance time was evaluated as surrogate for capsule opening. The caffeine appearance times can also be found in Table 3. None of the grade H and none of the grade M capsules showed premature caffeine appearance while capsules still resided in stomach. Only one grade L capsule already showed caffeine appearance in saliva during gastric residence, but this one also disintegrated visibly in MRI inside the stomach (see Fig. 7).

HPMCAS grade L test capsules showed significantly shorter dispersion and salivary caffeine appearance times than their co-administered HPMCAS grade H reference capsules. Same accounted for HPMCAS grade M test capsules, which showed significantly shorter dispersion and salivary caffeine appearance times than their co-administered HPMCAS grade H reference capsules too. There has been no significant difference between the two HPMCAS grade H reference capsules and also no significant difference between the two test capsules, administered at two different appointments. Nonetheless, the dispersion times after gastric emptying of HPMCAS grade H reference capsules showed a difference of the means of 18 min, while difference of the medians amounted to 8 min.

**Fig. 5:**
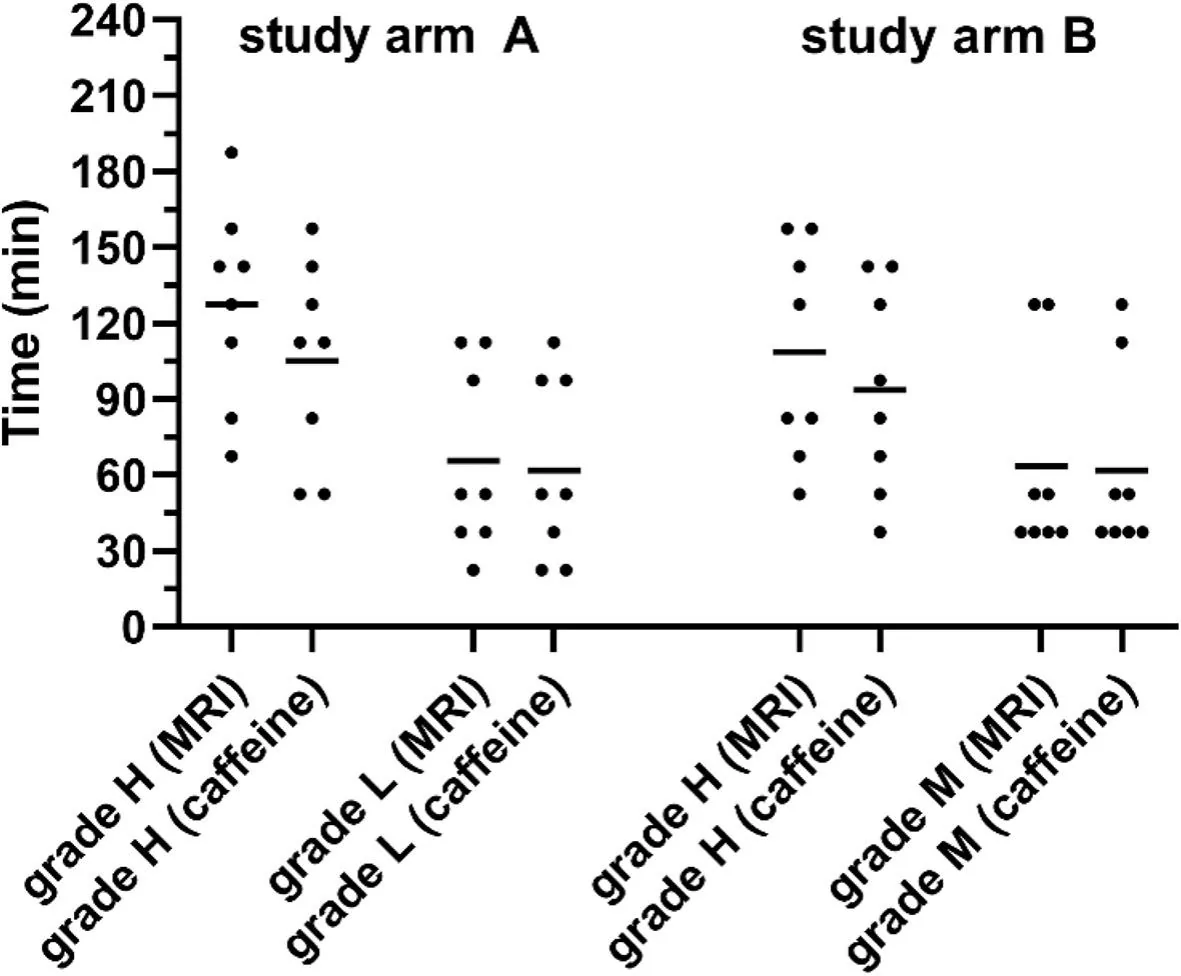
Comparison of Dispersion time from MRI and appearance time from salivary caffeine with median line (*n* = 8 each).

No significant difference in gastric emptying time were observed, neither within a study arm (test vs. reference), nor between the different applications (reference vs. reference). Nonetheless, GRT showed a remarkable variability not only between the subjects (interindividual), but also within the subjects (intraindividual). For example subject 2 had a GRT of its HPMCAS grade H capsule of 113 min in study arm A, whereas in study arm B it only amounted to 23 min. Subject 6 had a GRT of its HPMCAS grade H capsule of 23 min in study arm A, whereas it amounted to 143 min in study arm B. On the other side, subjects 1, 7 and 8 had identical GRTs between their respective study treatments. For a detailed evaluation of interindividual and intraindividual variability, sample size have number repetitions have been too small, but his has not been an aim of this study. Furthermore, the difference between imaging and pharmacokinetic labels as well as the difference between sensitivity of labeling techniques should be investigated. Slight differences between visual dispersion as detected by MRI and appearance of caffeine in saliva have been present throughout all investigated capsule types and study arms as can be seen in Fig. 5. After Bonferroni correction, no significant difference between dispersion time and appearance time in any of the capsules was observed, although a slight offset between visualized dispersion in MRI and caffeine appearance is visible for reference grade H capsules labeled with iron oxide. As can further be seen in Fig. 5, the difference between MRI dispersion and caffeine appearance seemed to be smaller for the test capsules, which might be attributed to different labeling technique.

**Fig. 6:**
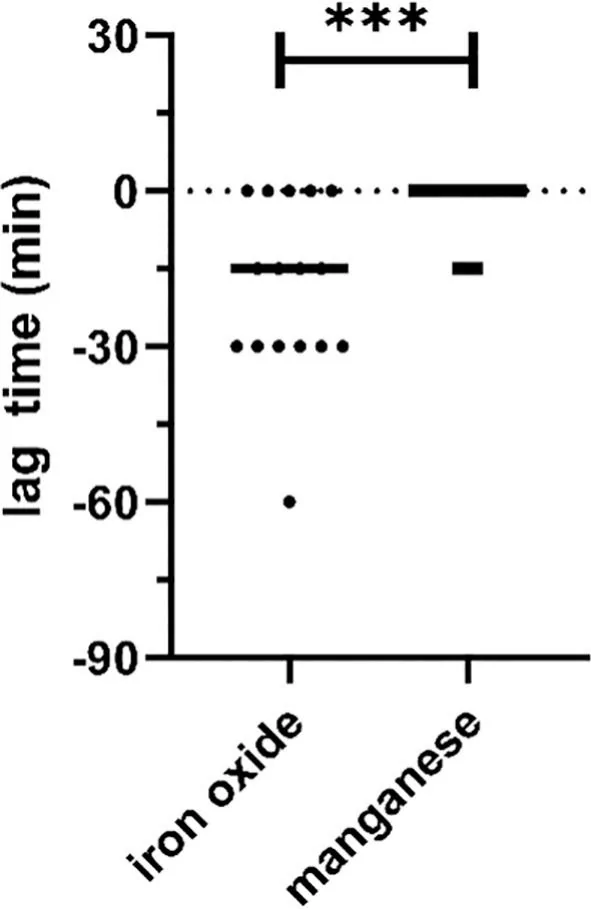
Lag time (difference between Dispersion time from MRI and appearance time from salivary caffeine) as evaluated by iron oxide dispersion or manganese signal appearance, with * significant difference according to Wilcoxon matched-pairs signed rank test (*n* = 16 each).

To investigate the sensitivity of imaging label for capsule opening, the difference between visible dispersion and salivary caffeine appearance was calculated as “lag time” and compared by imaging label. As can be seen in Fig. 6, the absolute value of the Lag time of iron oxide labeled capsules was significantly longer (*p* = 0.0020) than the lag time of manganese labeled capsules.

## 4 Discussion

In the present study, it was the aim to compare the in vivo performance of enteric capsule shells made of different grades of HPMCAS. Moreover, it has been the aim to establish a novel dual labeling method for MRI to track and distinguish two dosage forms in parallel, allowing for simultaneous application of reference and test formulation plus ideally improving also sensitivity of detection of release.

Besides the common techniques of dosage form labeling with black iron oxide for tracking in MRI and evaluation of release by salivary caffeine appearance, the utilization of manganese gluconate as secondary imaging label inside the test capsules offered excellent detectability of release by use of T1 weighted MRI.

**Fig. 7:**
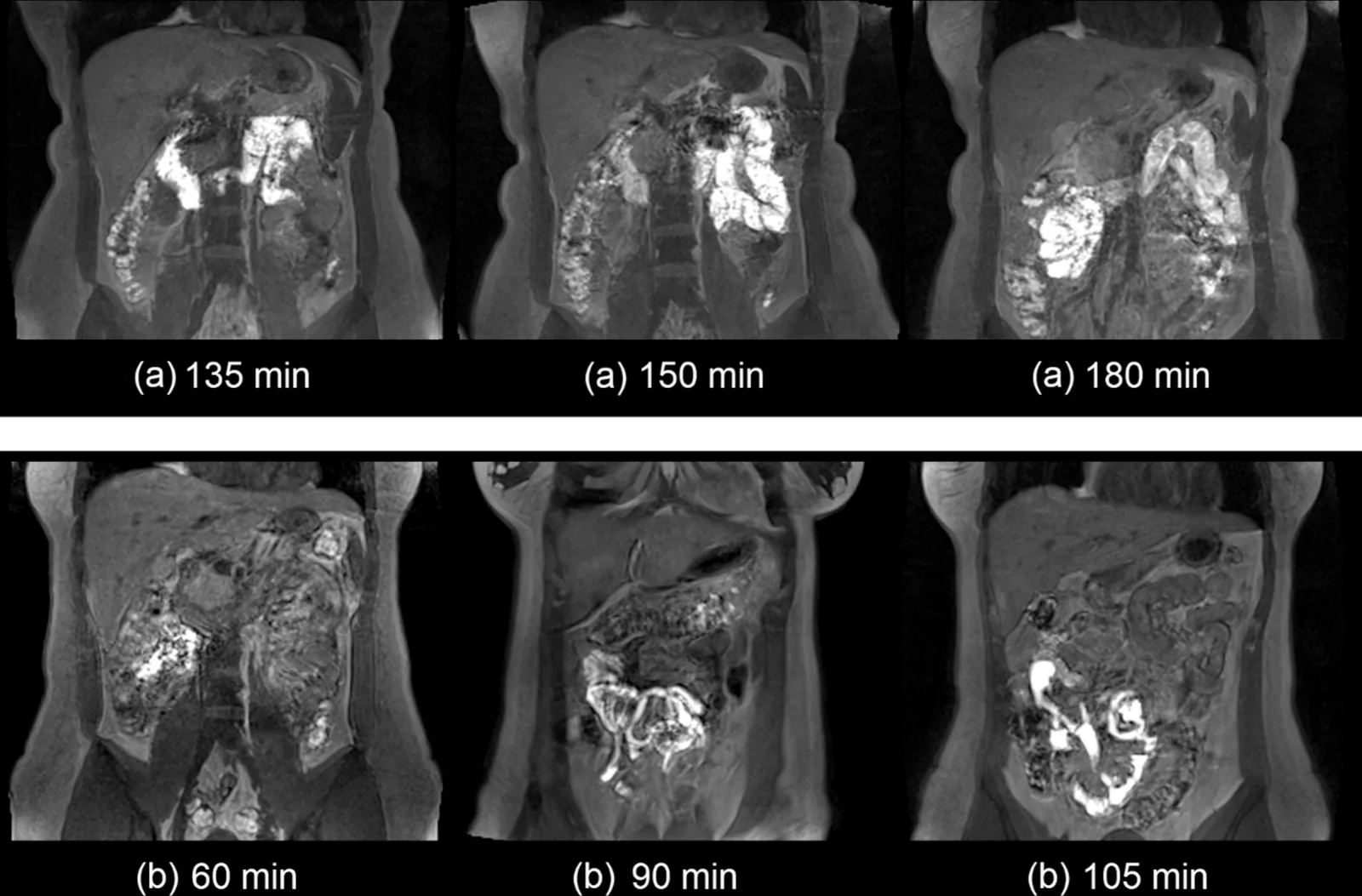
Intestinal spreading of water soluble label manganese gluconate after opening of enteric capsules in coronal T1 weighted VIBE maximum intensity projections with 5 stacked slices (a) and 3 stacked slices (b) in two exemplary subjects.

After opening of the test capsules, we observed a rapid increase in bright areas from manganese, and thus rapid distribution of the labeled filling throughout the small intestine, which might not be expected to this extend from previous MRI studies with insoluble markers (Rump et al., 2022; Grimm et al., 2023; Sager et al., 2019a; Rump et al., 2021). If also retrograde transport and dispersion occurred, could not be precluded from the images. But in general, mainly antegrade transport was observable. Since disintegration often took place in jejunum or proximal ileum, this would not be surprising. Although it is known that segmental, or mixing retrograde flow can occur, the proximal small intestine shows more propulsive motility (Seidl et al., 2012; Kerlin and Phillips, 1982; Kerlin et al., 1982). Exemplary images are given in Fig. 7.

It seems that the more proximal the disintegration took place, the more pronounced was subsequent spreading. With regards to regional motility in the small intestine this could be expectable. Since the proximal small intestinal regions typically show stronger and more frequent propulsive contractions and more transport of fluid to distal regions (Seidl et al., 2012; Kerlin and Phillips, 1982; Kerlin et al., 1982), subsequent spreading is promoted compared to disintegration in distal ileum. A recent patent also describes reduced dispersion of the released material in the distal and terminal ileum compared to jejunum. This has also been attributed to the lower motility in these regions (Tanguay et al., 2021). Similarly, less dispersion of a fluoroscopic marker was observed in the distal small intestine compared to proximal regions in dogs (Lee et al., 2000).

Nonetheless, in the present study these observations could not be quantified throughout all subjects and time points as imaging technique was not optimized for evaluation of intestinal volumes and reference capsules have not been labeled with manganese. This would need to be verified in future studies, but the manganese gluconate filling turned out to be not only a sensitive marker for solid dosage form disintegration but also a suitable model for intestinal dispersion of water soluble drug substances. Anyway, a more pronounced spreading after disintegration in more proximal regions would be logical, since more length and surface is available during subsequent transit of fluid and/or dispersed filling. This shows, that site-specific release from a targeted dosage form at a specific site in small intestines will deliver the drug substance not only to the localization of disintegration but also to deeper sections.

This could have further implications for absorption from enteric dosage forms with critical drugs. The more distal in small intestine disintegration occurs, the less surface might be available subsequently, which might limit bioavailability. Moreover, majority of intestinal fluids is typically localized in distal regions of small intestine (Schiller et al., 2005; Mudie et al., 2014), so that more volume might be available for the filling to be dispersed in, compared to more proximal disintegration. Substances with poor solubility might benefit from more solubilized drug substance, whereas lower concentrations could lead to impaired absorption. In the available salivary caffeine pharmacokinetics, no such trends have been observable. But due to high solubility and excellent permeability, caffeine is not a good model drug for such questions, so that further studies would be necessary to gather deeper insights into the relation of absorptive area and concentration effects in small intestine as it was already highlighted elsewhere (Grimm et al., 2018a; Funai et al., 2022; Großmann et al., 2024). Enteric capsules with different site of release like tested in the present study might be an interesting tool for evaluation of those questions. Moreover, such capsules could be interesting for the evaluation of absorption windows.

The time between opening or disintegration of a dosage form which is tracked by an imaging procedure and the appearance of a pharmacokinetic marker was referred as “Lag time” in the past. After intragastric disintegration of a dosage form, it takes a while until the drug substance is solubilized, distributed intragastrically and emptied from the stomach, so that it can be absorbed. Thus, a positive lag time is observed between visual intragastric disintegration and appearance of pharmacokinetic markers like caffeine as it represents the observed “lag” between disintegration and appearance (Rump et al., 2022; Sager et al., 2019b). On the other hand, small intestinal disintegration can lead to negative lag times, since appearance of the pharmacokinetic marker can be earlier than the observed disintegration or opening of a dosage form. In dependence of the capabilities of the imaging technique and used labels, drug substance can be released before the loss of structural integrity of the dosage form can be visualized. For the evaluation and tracking of enteric dosage forms, the appearance of the pharmacokinetic marker can also be regarded as reference for evaluation of sensitivity of an imaging procedure to detect opening, disintegration or release from a dosage form. This is also supported by an exemplary pharmacoscintigrafic study (Stevens et al., 2002). Our previous publications have shown immediate appearance of caffeine in saliva, whenever dissolved caffeine was presented to the small intestine, which is also the reason that this method can be used to evaluate gastric emptying, irrespective if measured in blood or saliva (Sager et al., 2019a; Senekowitsch et al., 2026; Sager et al., 2018). In our case, the appearance of the pharmacokinetic label caffeine and its isotope labeled counterparts is estimated to be more sensitive to initial rupture than MRI. This was proven in several previous studies when black iron oxide was used to track the dosage form and its dispersion was used to evaluate opening or disintegration (Rump et al., 2022; Grimm et al., 2023; Sager et al., 2019a).

The t_max_ of caffeine after fasted administration of our enteric capsules amounted to 105 ± 54 min (*n* = 8) for grade L, 99 ± 62 min (*n* = 8) for grade M and 147 ± 50 min (*n* = 16) for grade H respectively. The administration of enteric capsules obviously led to a prolongation compared to caffeine solutions with a t_max_ of 30 min (*n* = 10) (Blanchard and Sawers, 1983a), 33 ± 30 min (*n* = 8) (Blanchard and Sawers, 1983b) or 29 ± 10 min (*n* = 12) (Tzakri et al., 2023). Immediate release capsule administration with 50 mg of caffeine exemplarily led to t_max_ mean value of 85 ± 54 min (*n* = 12) (Kamimori et al., 2002).

In contrast to iron oxide labeling, the evaluation of opening by T1 weighted MRI with manganese gluconate showed nearly perfect alignment with salivary caffeine appearance with a t_lag_ of 0 min in 13 out of 16 subjects and turned out to be much more sensitive than the evaluation of iron oxide dispersion. Moreover, this technique also allows for evaluation of release and not only disintegration e.g. from extended release dosage forms as recently also considered by Akbar et al. (Akbar et al., 2024b). In general this approach is comparable to the fundamental work by Steingoetter et al., evaluating the release from tablets by use of gadolinium (Steingoetter et al., 2003) but avoiding the health risks and regulatory effort that would result from the use of this contrast agent.

Although black iron oxide allows easy tracking, its limited sensitivity for initial release from dosage forms and its disturbance of imaging due to pronounced artifacts led to the wish of other labeling techniques. In the past we have made several attempts for a dosage form label, trackable in T1 weighted imaging and avoiding black iron oxide.

Dried and sugared pineapple pieces have been clearly visible and allowed for tracking of sinking and floating capsule in capsule formulations, but differences in pineapple products and batches in terms of residual moisture and manganese content avoided further broad application. Moreover, residual moisture hindered representative applicability to some dosage forms and addition of pharmacokinetic label (Grimm et al., 2019). For visualization of esophageal films hibiscus tea extract rich in manganese was successfully used (Rosenbaum et al., 2021), but implementation of hibiscus tea powder to DUOCAP® capsule in capsule formulations did not allow robust evaluation of opening according to bright signal in T1 weighted MRI (Rump et al., 2021). In another study with enteric capsules, these capsules have been filled 10 mg black iron oxide, 87.5 mg manganese gluconate, 75 mg natural caffeine powder and topped up with sugar pellets. Capsules have been trackable due to iron oxide, but bright manganese cloud was most often not detected. Most likely it has been overshadowed by the excessive amount of black iron oxide and its susceptibility artifacts or the dose of manganese gluconate has been inadequate (Grimm et al., 2024). Since black iron oxide is insoluble in gastrointestinal media, the black artifacts persist, even after capsule opening.

In the present study, the test capsules have been labeled with only 2.2 mg black iron oxide for tracking and 125 mg manganese gluconate and 25 mg ^13^C_1_ labeled caffeine for evaluation of opening. The appearance of manganese signal offered very sensitive evaluation of capsule opening in all cases. This was most likely attributed to the reduced amount of iron oxide leading to much smaller susceptibility artifacts after opening and an increased amount of manganese gluconate. Moreover, croscarmellose was incorporated which might provoke more pronounced initial spreading and formation of a bright cloud.

**Fig. 8:**
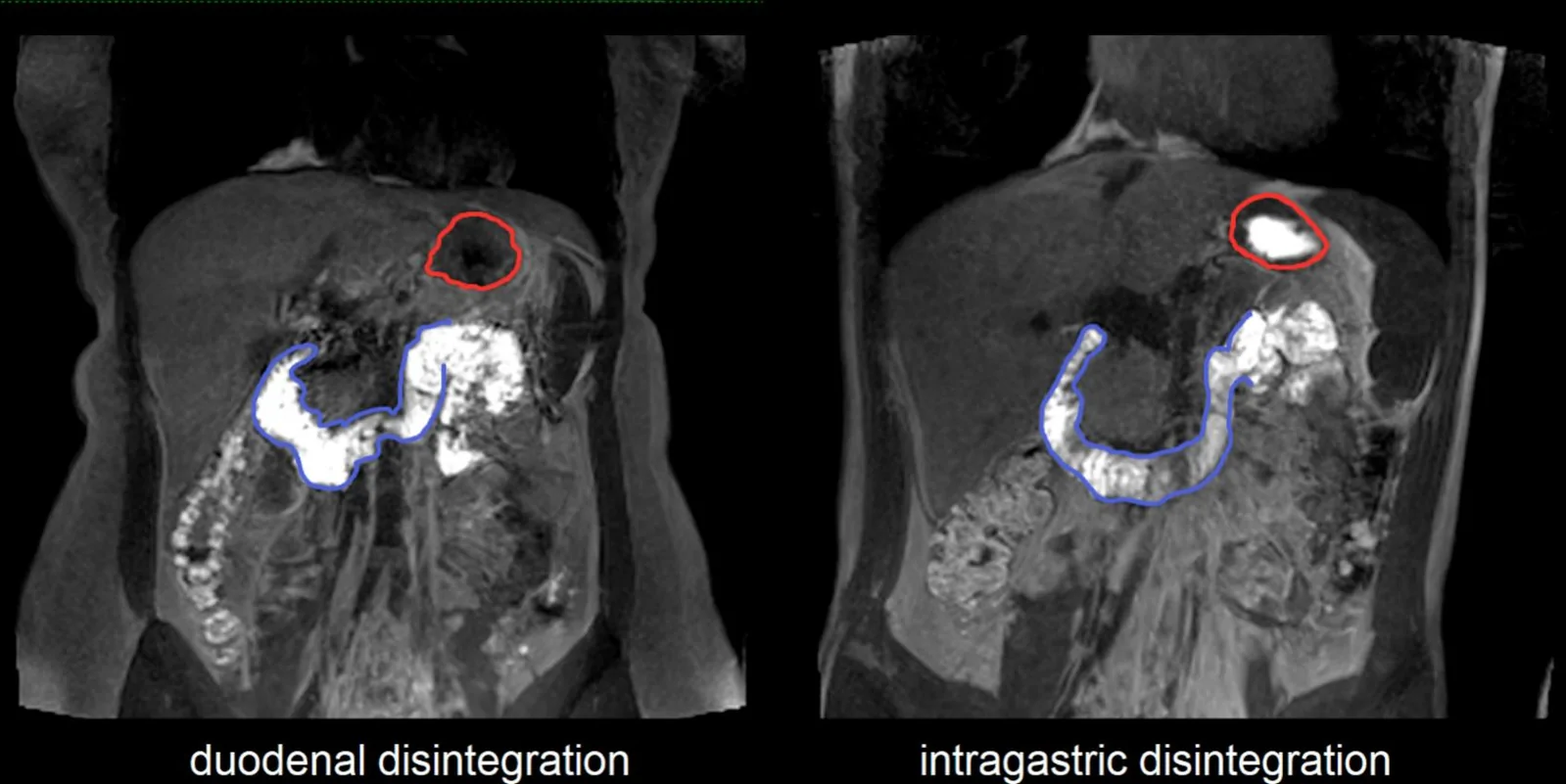
Bright clouds of released manganese from enteric capsules in coronal T1 weighted VIBE maximum intensity projections (6 stacked slices) allowing discrimination between duodenal disintegration (left) and premature intragastric disintegration (right) with stomach marked red and duodenum marked blue. (For interpretation of the references to colour in this figure legend, the reader is referred to the web version of this article.)

Thus, the manganese labeling allowed for very sensitive detection of leakages of the capsules. It is obvious, that most often the opening of the capsules took place between two imaging procedures and not directly at an imaging time point, as the interval between measurements was 15 min. If a capsule resides in the stomach at one time point and at the next time point it is detected as opened in the small intestine, it could not be robustly distinguished were release started by the use of black iron oxide label. Detectable amounts of iron oxide do not necessarily stay inside the stomach or are not even released at this step. A detectable amount of iron oxide could also be emptied with the broken capsule residues. As only dispersed artifacts in small intestine will be observed, it is not distinguishable whether release already took place in stomach or not. The manganese label dramatically improves the sensitivity for premature leakages, as even small amounts of released manganese will lead to an observable contrasting effect. Also homogeneity is better since a solution is formed rapidly allowing for easy mixing and diffusion. Since an enteric dosage form should ideally provide no release inside the stomach, this detectability is of decisive interest for the evaluation of the tested enteric HPMCAS capsules. The appearance of bright signal inside the stomach enabled good discrimination between intragastric and duodenal opening as can be seen in Fig. 8. In the respective example of premature intragastric release, the iron oxide label of the test capsule just disappeared and caffeine appeared in saliva, but it would have remained unclear were exactly this process started. The residing manganese solution inside the stomach undoubtedly showed that the HPMCAS grade L capsule failed regarding its enteric properties.

For determination of appropriate amount of manganese gluconate it needs to be considered that the amount of available fluid for solubilization can differ dramatically in the small intestines where the capsules were aimed to disintegrate. A capsule can be in contact with several milliliters of intestinal media but they also cannot have contact with free fluid at all as reported by Schiller et al. (Schiller et al., 2005). Thus, very different concentrations of manganese inside the media can result. For evaluation of opening by its action as MRI contrast agent, a sufficient concentration is needed, without exaggerate local amounts of manganese ions, since these could again lead to a signal loss. Excessively high concentrations of manganese due not only shorten T1 relaxation time which would lead to high signal in T1 weighted imaging, but also excessively shorten T2 and T2* relaxation, which can cause rapid dephasing of proton spins, leading to a loss of signal even in T1 weighted imaging. After several pilot experiments, 125 mg manganese gluconate turned out to be an optimal amount for size 0 capsules, facilitating detection of capsule opening in all investigations with very good agreement with pharmacokinetic appearance of caffeine from the capsule filling.

Nonetheless, due to 3D acquisition within up to 18 s, the used VIBE sequence for detection of manganese clouds is prone to movement artifacts from small intestinal motility and same might account for equivalent simple sequences of other manufacturers of MRI devices e.g. LAVA or THRIVE. This was mainly attributed to the use of an older Siemens MAGNETOM Avanto with 1.5 T in this study. Faster T1 weighted imaging with improved acceleration techniques might reduce these issues allowing more precise measurement e.g. of manganese cloud volumes or contact surface of a simulated API cloud. On the other side, this study shows applicability of the manganese labeling technique even with standard sequences on 1.5 T.

The tracking of dosage forms by the use of black iron oxide is a well-described technique and has been utilized for many years (Steingoetter et al., 2003; Knörgen et al., 2010; Kagan et al., 2006). Nonetheless, discrimination of individual simultaneously taken dosage forms was not performed until now. By use of very different amounts of black iron oxide in the powder filling of the enteric capsules, two capsules could be distinguished successfully in the recent study.

Discrimination between test and reference capsule by size of susceptibility artifact worked well, with only very few exceptions when both capsules have been so close together that the large artifact of the reference capsule overshadowed the smaller artifact of the test capsule. This sometimes occurred when both capsules resided in a relatively empty stomach.

Theoretically, capsule orientation in imaging plane and size of cross-section play a crucial role in distinctiveness. If both capsules are imaged along their long side (lying flat in the plane), so that the imaging plane hits them across their largest dimension, the artifacts are distinguishable due to their different sizes arising from different amounts of iron oxide in the imaging plane. But the capsules are able to move freely in the GIT and especially in small intestine it is not unlikely that capsules are imaged across their smallest dimension when they are orientated upright in the plane. This would reduce visible artifact size within this plane and could lead to an artifact of the highly labeled capsule of similar size that the lower labeled capsule. We have seen this effect, but capsules stayed distinguishable with the used amounts of iron oxide. Nonetheless, in general the distinctiveness could be affected by capsule orientation.

For tracking of dosage forms iron oxide is superior to manganese gluconate, because even small amounts allow identification of the dosage form with its dry core. The technique is therefore also able to detect gastric emptying and thus allows for determination of GRT of solid oral dosage forms. The GRT is relevant for enteric dosage forms in particular, since it strongly determines the time of drug release after intake.

The pronounced variability in GRT observed in this study is not surprising and already described in other publications on dosage form tracking (Grimm et al., 2019; Schneider et al., 2016; Davis et al., 1986; Park et al., 1984). It is well known, that solid monolithic dosage forms typically need propulsive motility and an opened pylorus associated with phase III of the interdigestive migrating motor complex (MMC). Otherwise, they tend to rest inside the stomach since a sufficient force is missing to pass the closed or only slightly opened lower gastric sphincter. The MMC is a repetitive motility pattern present in the upper gastrointestinal tract during fasted state. Besides phase I and II which exhibit motoric resting state or low contractions, so-called housekeeping waves appear in stomach during phase III. Since the emptying of solid monolithic dosage forms is mainly depended on these housekeeping waves and thus also depended on the motility pattern in which subjects are after 10 h of fasting, the gastric emptying of these objects is highly erratic (Koziolek et al., 2013; Koziolek et al., 2016).

Only in rare cases, even monolithic dosage forms like capsules are emptied immediately after intake (Rump et al., 2022; Grimm et al., 2019; Wilding et al., 1992; Schneider et al., 2016). It remains unclear, whether they are flushed out through the opened pylorus with the water flow or if subjects are in phase III of MMC by chance during dosage form application.

With 18 min difference, mean dispersion times after gastric emptying of Capsugel® Enprotect® capsules differed considerably between the two study arms. Although not significant, this was surprising as same subjects, same capsules from same batch, same filling and same setting have been used. In theory this could also be attributed to variability of the capsule shell itself, but we consider this rather unlikely as physiological variability is known to be high and no hints for variable capsule performance where observed in previous human studies (Rump et al., 2022; Grimm et al., 2023) or in vitro (Jannin et al., 2023). This shows that even in crossover study designs considerable differences after repetitive administration can occur and highlights the superior study design with an internal reference which allowed us to compare test and reference capsules in the same administration without the effect of intraindividual variability. Such intraindividual variability is known for residual gastric content volumes (Roelofs et al., 2024; Grimm et al., 2018b) and thus it might also be relevant for the starting pH of gastrointestinal transit with effects on subsequent polymer erosion from the capsule shell.

As estimated, the utilization of lower substituted HPMCAS grades led to a shortening in opening time and to a shift in capsule opening to more proximal sections of the GIT. Capsules made of HPMCAS grade M disintegrated predominantly in the jejunum with robust withstanding the gastric transit in our small cohort. This is a valuable step towards delivery to more proximal intestinal regions by use of enteric capsules. Taken into account the known pH profiles in small intestine e.g. from telemetric capsule studies (Schneider et al., 2016; Koziolek et al., 2015) with mean and median pHs in proximal small intestine of mainly around pH 6, this could be estimated for a polymer which starts to dissolve at this pH.

On the other side, capsules made of HPMCAS grade L also shifted capsule opening to more proximal sections compared to HPMCAS grade H capsules. But in one subject the capsule opening already took place inside the stomach, which needs to be regarded as a fail of the enteric system. This might again be related to mechanical stress in the antral area. But moreover, the starting pH in the stomach after ingestion of such capsules together with a glass of water can be higher than pH 4 and up to pH 6.4 which was observed in a SmartPill® study (Funai et al., 2022). Also Intellicap® telemetric capsule data show transient intragastric pH values above pH 5 in several subjects during gastric residence (Großmann et al., 2024). Since HPMCAS grade L dissolves at pH ≥ 5.5, these high intragastric pH values could cause preliminary erosion of the shell, so that it was not able to withstand the antral mechanical stress anymore. Although this event seems not very likely (one out of eight administrations), the risk of failure when using this polymer type for enteric dosage forms seems relevant. Irrespective of these results, for the use of HPMCAS grade L in amorphous solid dispersions, these solubility properties could even be beneficial, depending on drug substance and formulation method that is going to be used.

Since size, shape and shell wall thickness have been the same and capsule filling has been a very similar and representative powder mixture, the observed effects are most likely related to the different polymer grades and no other factors, except physiology.

## 5 Conclusions

Capsugel® Enprotect® capsules made of HPMCAS grade H were proven again to provide robust enteric properties with disintegration mainly taking place in ileum. As estimated, lowering the substitution grade of used shell polymer HPMCAS to grade M shortened the time to disintegration after gastric emptying, leading to more proximal disintegration in small intestine. In contrast, tested capsules made of HPMCAS grade L disintegrated even more proximal, but also failed in one subject showing gastric release.

The dual labeling allowed to compare the test capsules with the reference capsules as internal standard within the same application, increasing statistical robustness by avoidance of intraindividual variability and allowing for an efficient study design with less subjects and less individual study arms. Moreover, manganese labeling of capsules allowed for interesting insights into intestinal dispersion of a water soluble model drug after disintegration of an enteric dosage form and further turned out to be more sensitive than iron oxide labeling to detect beginning release from capsules. Nonetheless, for tracking of intact solid oral dosage forms with dry substance, low amounts of iron oxide or other trackable components remain necessary.

## CRediT authorship contribution statement

**Michael Grimm:** Writing – original draft, Supervision, Project administration, Methodology, Investigation, Formal analysis, Conceptualization. **Ruben Lau:** Visualization, Methodology, Investigation, Formal analysis. **Fiona Mankertz:** Writing – review & editing, Resources, Investigation. **Robin Bülow:** Writing – review & editing, Resources, Investigation. **Felix Morof:** Validation, Formal analysis. **Delphine Nombret:** Resources, Funding acquisition. **Camille Dumont:** Writing – review & editing, Funding acquisition. **Vincent Jannin:** Writing – review & editing, Supervision, Funding acquisition. **Mladen Vassilev Tzvetkov:** Writing – review & editing, Supervision, Resources. **Werner Weitschies:** Writing – review & editing, Supervision, Resources, Funding acquisition, Conceptualization.

## Informed consent statement

Informed consent was obtained from all subjects involved in the study. Subjects are not identifiable in these data; nonetheless, written informed consent has been obtained from the subjects to publish this paper.

## Institutional review board statement

The study was conducted according to the guidelines of the Declaration of Helsinki, and approved by the Ethics Committee of the University Medicine of Greifswald (ethical protocol No. BB 115/24a). The study was registered at the German Register for Clinical trials under DRKS00035518.

## Ethics declaration

Written informed consent to take part in the study and to publish the article has been obtained from all participants or their legal representatives. The privacy rights of participants have been observed.

This study was performed in compliance with relevant laws, regulatory frameworks and guidelines where the research took place.

This study was approved by the Ethical Committee at the University Medicine Greifswald.

(Approval No. BB 115/24a)

## Funding

This research was funded by Capsugel France SAS (Colmar, France).

## Declaration of competing interest

The authors declare the following financial interests/personal relationships which may be considered as potential competing interests: Werner Weitschies reports financial support was provided by Capsugel Colmar. Camille Dumont reports a relationship with Capsugel Colmar that includes: employment. Delphine Nombret reports a relationship with Capsugel Colmar that includes: employment. Vincent Jannin reports a relationship with Capsugel Colmar that includes: employment. If there are other authors, they declare that they have no known competing financial interests or personal relationships that could have appeared to influence the work reported in this paper.

## Data Availability

Data will be made available on request.
