# Supplementary material for: In vivo comparison of enteric capsule shells composed of different HPMCAS grades after simultaneous intake by dual MRI labeling and pharmacokinetics
Source: Int J Pharm X. 2026 Sep 15;12:100666. doi: 10.1016/j.ijpx.2026.100666 (PMC13599660; doi:10.1016/j.ijpx.2026.100666)
Supplement: Supplementary file 1 [file mmc1.docx]

**Supplementary Data on:**

**In vivo comparison of enteric capsule shells composed of different HPMCAS grades after simultaneous intake by dual MRI labeling and pharmacokinetics**

Michael Grimm ^1.^*, Ruben Lau ^1^, Fiona Mankertz ^2.3^ , Robin Bülow ^2^, Felix Morof ^4^, Delphine Nombret ^5^, Camille Dumont ^5^, Vincent Jannin ^5^, Mladen Vassilev Tzvetkov ^4^, Werner Weitschies ^1^

^1^	Department of Biopharmaceutics and Pharmaceutical Technology, University of Greifswald,

17489 Greifswald, Germany

^2^	Department of Diagnostic Radiology and Neuroradiology, University Hospital Greifswald,

17475 Greifswald, Germany

^3^	Department of Radiology, University Hospital Tuebingen, 72076 Tuebingen, Germany

^4^	Department of General Pharmacology, University Hospital Greifswald. 17487 Greifswald, Germany

^5^	Capsugel France SAS, 68000 Colmar, France

** Corresponding author:*

Dr. Michael Grimm

Department of Biopharmaceutics and Pharmaceutical Technology. Center of Drug Absorption and Transport. University of Greifswald. Greifswald. Germany

Felix-Hausdorff-Str. 3. D-17487 Greifswald. Germany

Tel +49 3834 420 4816

[michael.grimm@uni-greifswald.de](mailto:michael.grimm@uni-greifswald.de)

# Supplementary Table S1: Individual Gastric Residence Times

|  | study arm A | | study arm B | |
| --- | --- | --- | --- | --- |
| **Subject** | **grade H** | **grade L** | **grade H** | **grade M** |
| 001 | 7.5 | 7.5 | 7.5 | 7.5 |
| 002 | 112.5 | - | 22.5 | 22.5 |
| 003 | 52.5 | 52.5 | 52.5 | 22.5 |
| 004 | 37.5 | 22.5 | 7.5 | 7.5 |
| 005 | 7.5 | 22.5 | 7.5 | 37.5 |
| 006 | 22.5 | 22.5 | 142.5 | 127.5 |
| 007 | 22.5 | 22.5 | 22.5 | 22.5 |
| 008 | 82.5 | 97.5 | 82.5 | 97.5 |

# Supplementary Table S2: Individual Dispersion Times

|  | study arm A | | study arm B | |
| --- | --- | --- | --- | --- |
| **Subject** | **grade H** | **grade L** | **grade H** | **grade M** |
| 001 | 82.5 | 37.5 | 67.5 | 37.5 |
| 002 | 187.5 | 37.5 | 82.5 | 52.5 |
| 003 | 157.5 | 97.5 | 142.5 | 37.5 |
| 004 | 112.5 | 22.5 | 82.5 | 37.5 |
| 005 | 127.5 | 112.5 | 52.5 | 37.5 |
| 006 | 67.5 | 52.5 | 157.5 | 127.5 |
| 007 | 142.5 | 52.5 | 127.5 | 52.5 |
| 008 | 142.5 | 112.5 | 157.5 | 127.5 |

# Supplementary Table S3: Individual Dispersion Times after Gastric Emptying

|  | study arm A | | study arm B | |
| --- | --- | --- | --- | --- |
| **Subject** | **grade H** | **grade L** | **grade H** | **grade M** |
| 001 | 75 | 30 | 60 | 30 |
| 002 | 75 | - | 60 | 30 |
| 003 | 105 | 45 | 90 | 15 |
| 004 | 75 | 0 | 75 | 30 |
| 005 | 120 | 90 | 45 | 0 |
| 006 | 45 | 30 | 15 | 0 |
| 007 | 120 | 30 | 105 | 30 |
| 008 | 60 | 15 | 75 | 30 |

# Supplementary Table S4: Individual Salivary Caffeine Appearance Times

|  | study arm A | | study arm B | |
| --- | --- | --- | --- | --- |
| **Subject** | **grade H** | **grade L** | **grade H** | **grade M** |
| 001 | 52.5 | 37.5 | 37.5 | 37.5 |
| 002 | 157.5 | 22.5 | 67.5 | 52.5 |
| 003 | 127.5 | 97.5 | 142.5 | 37.5 |
| 004 | 112.5 | 22.5 | 82.5 | 37.5 |
| 005 | 112.5 | 97.5 | 52.5 | 37.5 |
| 006 | 52.5 | 52.5 | 142.5 | 127.5 |
| 007 | 82.5 | 52.5 | 97.5 | 52.5 |
| 008 | 142.5 | 112.5 | 127.5 | 112.5 |
